# Classroom intervention to improve behavioral intention toward regular physical activity among adolescents

**DOI:** 10.3389/fpubh.2024.1260916

**Published:** 2024-08-07

**Authors:** Sweety Suman Jha, Madhumita Dobe, Chandrashekhar Taklikar, Arista Lahiri

**Affiliations:** ^1^Community Medicine, Dr. B.C. Roy Multi-Speciality Medical Research Centre, Indian Institute of Technology Kharagpur, West Bengal, India; ^2^Department of Health Promotion & Education, All India Institute of Hygiene and Public Health (AIIH&PH), Kolkata, West Bengal, India

**Keywords:** adolescents, behavioral intention, intervention, non-communicable disease, physical activity

## Abstract

**Background:**

During adolescence, a critical phase in human life, the groundwork for a healthful future is established. Physical inactivity poses a significant risk factor for non-communicable diseases (NCDs) and related mortality worldwide. To assess adolescents’ behavioral intentions regarding regular physical activity, the Theory of Planned Behavior (TPB) examines ‘Attitude,’ ‘Subjective norm,’ and ‘Perceived behavioral control.’ Utilizing TPB, this study focuses on evaluating the impact of a school-based health promotion intervention on behavioral intentions toward physical activity among urban adolescents in West Bengal, India.

**Methods:**

A school-based nonrandomized controlled interventional study with parallel group design was conducted among adolescents aged between 12 and 16 years. Behavioral intention towards performing regular physical activity was determined with the measurements of the constructs from the TPB. Cluster analysis was conducted using measurements from both the intervention and control groups. Participants with higher mean scores in the constructs were classified as intenders, while the rest were considered non-intenders. The intervention’s impact was evaluated by calculating the Relative Risk (RR) through a generalized linear model with robust standard error estimates, to ascertain the probability of belonging to the higher intention cluster.

**Result:**

Following the intervention, construct-wise scores improved significantly, particularly the perceived behavioral control mean score in the intervention group. The Relative Risk (RR) of becoming an intender for regular physical activity in the intervention group was 1.24 (95% CI: 1.04–1.48) when compared to the control group.

**Conclusion:**

Health Promoting Schools has been recognized as a strategic and cost-effective vehicle to promote positive development and healthful living, and the current evidence suggests they can effectively reduce the emergence of significant NCD risk factor like physical inactivity. Schools must establish strong partnerships with diverse stakeholders to address barriers beyond the school environment and enhance their control over critical issues.

## 1 Background

The World Health Organization (WHO) and the United Nations (UN) have defined adolescents as individuals in the age group of 10–19 years (1). This stage of life is marked by intense physical, psychological, and cognitive development (2). Adolescence is a pivotal phase that builds upon the developments of the first decade of life, guiding individuals through risks and vulnerabilities, and propelling them towards realizing their full potential. Globally, the WHO estimates that there are nearly 1.2 billion adolescents aged 10–19 years (3), with some countries having them make up as much as one-fourth of their population. The number of adolescents is expected to increase, especially in low- and middle-income countries (LMICs), where almost 90% of them currently reside (4), with India alone accounting for approximately 243 million adolescents (5).

Physical activity encompasses various bodily movements that demand energy expenditure, including a diverse array of activities such as work, play, household chores, travel, and recreational pursuits (6–8). Insufficient physical activity, or physical inactivity, is a significant risk factor for non-communicable diseases (NCD) and premature death worldwide. Neglecting adequate levels of physical activity increases the risk of obesity, cancer, heart disease, stroke, and diabetes by more than 20%, leading to a shortened lifespan of around 3–5 years. The growing levels of overweight and obesity among children and adolescents are particularly concerning, as they are associated with increased morbidity risk in adulthood (9). Additionally, physical inactivity places a burden on society through the hidden and growing costs of medical care and lost productivity (6). Physical activity also exerts a notable influence on mental health. Studies in India have shown promising results, indicating that physical activity, including exercise and yoga, can effectively reduce mean scores for severe and common mental disorders (10).

A survey in India revealed that 54.4% of individuals were inactive, 31.9% were active, and 13.7% were highly active in terms of physical activity patterns (11). An interventional study among adolescents showed promising effects on increasing physical activity levels and improving physical activity knowledge (12). Another study demonstrated that a classroom-based intervention could enhance students’ average daily Moderate to Vigorous Physical Activity (13). These initiatives aim to increase awareness, expand knowledge, and develop skills, fostering a health-oriented attitude among individuals who are integral parts of society (14). However, changing behavior requires more than just knowledge. The Theory of Planned Behavior offers insights into the behavioral intentions of adolescents concerning regular physical activity. It assesses the following constructs: ‘Attitude,’ ‘Subjective norm,’ and ‘Perceived behavioral control’ (15).

In the Theory of Planned Behavior (TPB) (15, 16), several fundamental constructs influence human behavior. First, Behavioral Intention refers to the extent to which an individual formulates plans to achieve a specific behavioral goal—the perceived likelihood of actually performing the behavior. Attitude, another crucial aspect, involves considering the outcomes of a behavior and evaluating it favorably or unfavorably. Next, Subjective Norm reflects an individual’s belief about whether influential people in their life approve or disapprove of the behavior. This social influence plays a significant role in shaping decisions. The final construct, Perceived Behavioral Control (PBC), focuses on how a person perceives a particular behavior’s ease or difficulty. It encompasses the perceived likelihood of various conditions facilitating or constraining the behavior and their perceived impact on behavioral performance. Together, these four constructs form the foundation of the TPB, providing valuable insights into the complexity of human decision-making and behavior. Understanding these components empowers researchers and practitioners to design targeted interventions to promote positive outcomes in various domains, such as health, education, and social behavior. By comprehending the factors that influence behavior, we can pave the way for more effective and informed strategies to support individuals in achieving their goals and creating positive change in their lives.

Initiating behavioral interventions during adolescence holds considerable promise for cultivating a clear behavioral intention, given this stage’s formative impact on the human life cycle. With young children being receptive to behavioral corrections, adolescence presents an opportune time for preventive interventions against the development of risk factors leading to unhealthy behaviors in adulthood. Schools, providing a captive population of adolescents, serve as an ideal setting for the implementation and evaluation of health promotion interventions. Although there are a number of studies conducted on understanding of the practice of regular physical activity among the school-going adolescents (some involving health behavior theories), the examination of these different proposed techniques through appropriate interventional research is lacking (13, 17–32). Among the interventional studies conducted on similar topics, theory-based interventions are a rarity, and even those who conducted theory-based enquiry into the effect of health promotion interventions, did in terms of total score, while an estimation of intervention effect in terms of a person becoming intender or not is of utmost importance for policy and advocacy (13, 22, 24, 25, 29). To address this gap the current article focuses demonstrating the effect of health promotion intervention for improving the intention towards regular physical activity among the adolescents. In this context, our current study examined the change in in the proportion of intenders, as assessed through the Theory of Planned Behavior (TPB), with a focus on fostering regular physical activity among adolescents in an urban area of West Bengal, India.

## 2 Methods

### 2.1 Study design

A school-based nonrandomized controlled interventional study with parallel group design was conducted in two co-educational English medium schools in a selected municipal area of West Bengal. For the purpose of this study two schools in Uttarpara-Kotrung Municipal area in West Bengal, India were chosen based on enrolment and attendance, and an overall matching socio-demographic profile of the students. Non-random allotment of one school for the intervention arm and the other for control arm was done. The data collection was done between March 2019 to January 2020.

### 2.2 Study participants

**Figure 1:**
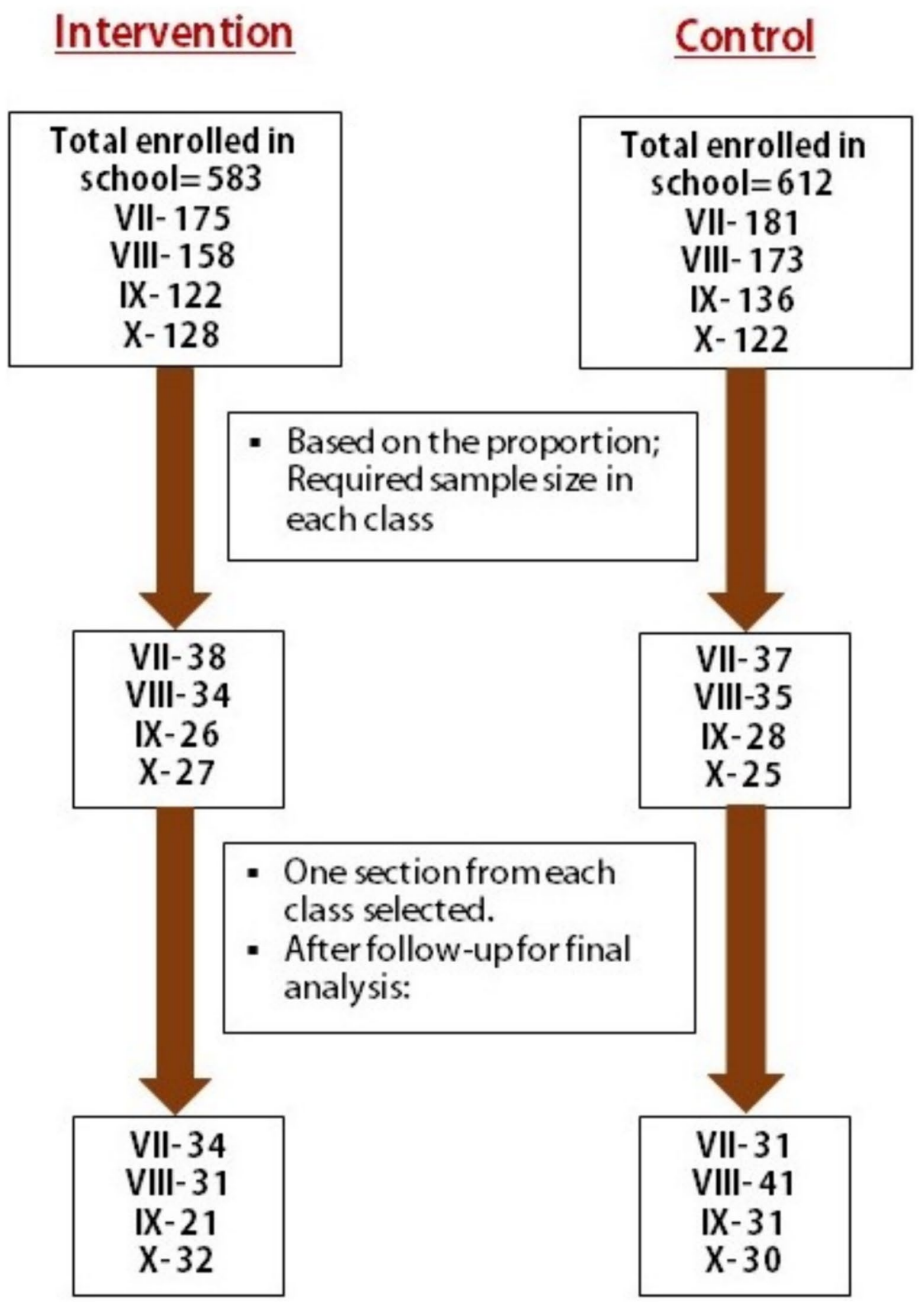
Selection of the participants.

Adolescents in seventh to tenth grades aged between 12 and 16 years, studying in the selected schools, whose parents gave consent and who provided assent for participation, were included in the study. However, those who were absent at any phase of the study were excluded from the study. In each of the selected schools, one section from each of the grades were chosen based on probability proportional to size, and then the students enrolled in those sections were recruited. The details of inclusion and exclusion criteria, and participant selection can be found elsewhere (33). Figure 1 shows the selection of study participants, where 133 completed responses in the control group and 118 completed responses in the intervention group were finally included on completion of the post-intervention survey. The socio-demographic characteristics of the participants are outlined in Table 1.

**Table 1:** Socio-demographic characteristics of the participants.

**Socio-demographic characteristics**		**Intervention (*n* = 118)**		**Control** **(*n* = 133)**		**Total** **(*n* = 251)**		***P*-value**
		***N***	**%**	***N***	**%**	***N***	**%**	
Age (in completed years)	12	9	7.63	10	7.52	19	7.57	0.329
	13	21	17.80	32	24.06	53	21.11	
	14	37	31.35	50	37.60	87	34.66	
	15	39	33.05	30	22.55	69	27.49	
	16	12	10.17	11	8.27	23	9.17	
Gender	Boys	80	67.80	66	49.62	146	58.17	0.004
	Girls	38	32.20	67	50.38	105	41.83	
Religion	Hinduism	111	94.06	126	94.74	237	94.42	0.974
	Islam	4	3.39	4	3.00	8	3.19	
	Others*	3	2.55	3	2.26	6	2.39	
Type of family	Nuclear	70	59.32	94	70.68	164	65.33	0.059
	Joint	48	40.68	39	29.32	87	34.67	

‘*n*’ indicates the total participants in respective groups, ‘*N*’ denotes the numbers in each category, and ‘%’ represents the respective column percentages. *p*-values are obtained by Chi-squared tests. *Others include Jainism and Sikhism.

### 2.3 Measurements

The measurement of behavioral intention towards regular physical activity employed constructs derived from the Theory of Planned Behavior (TPB), namely: (i) ‘Attitude,’ encompassing behavioral belief and evaluation of behavioral outcome, (ii) ‘Subjective norm,’ including normative beliefs and motivation to comply, and (iii) ‘Perceived behavioral control’ (PBC), comprising control beliefs and perceived power (16). To determine the items for construct measurement, elicitation interviews were conducted among 20 students from classes VII to X in a separate English medium co-educational school within the study area, adhering to the elicitation interview guide centered around TPB constructs.

Attitude was gauged using the following items: ‘Regular physical activity leads to the proper functioning of the body system,’ ‘Regular physical activity helps to boost energy level throughout the day,’ ‘Physical activity increases laziness,’ ‘Physical activity decreases sleeping time,’ ‘Physical activity increases hunger,’ and ‘Physical activity has no role in being stronger and taller.’ For Subjective Norms, the items encompassed ‘Mother,’ ‘Father,’ ‘Relatives/other family members,’ ‘Friends/peers,’ ‘Teachers,’ ‘Contents of television,’ and ‘Contents/discussions in social media.’ Regarding the PBC construct, the items included: ‘Choosing to perform regular physical activity even when there is no playground nearby,’ ‘Choosing to play outdoors despite homework and academic pressure,’ ‘Choosing to perform regular physical activity despite the thought that people may tease an obese person in a very embarrassing manner,’ ‘Choosing to perform regular physical activity even if playing outdoor games become difficult,’ ‘Choosing to play or do physical activity despite video games and/or mobile phone games,’ ‘Choosing to perform physical activity even during special days/occasions,’ and ‘Choosing to do regular physical activity/exercises despite laziness.’

Each item underwent assessment within all three constructs, employing dichotomous response options (Agree-Disagree or Likely-Unlikely). In the pre- and post-intervention phases, participants with higher intentions were identified through a combined evaluation of construct-wise measurements. This approach facilitated a robust evaluation of behavioral intention changes towards regular physical activity, enabling an informed understanding of the intervention’s impact on the study participants.

### 2.4 Instruments

To evaluate behavioral intention towards regular physical activity, a pre-designed, pre-tested, and validated questionnaire was employed. The questionnaire encompassed socio-demographic characteristics and the three fundamental constructs from the TPB, instrumental in determining the intention to engage in regular physical activity. The content of the questionnaire was extracted from the findings of the elicitation interviews conducted with the students comparable in terms of age and gender (from other schools) regarding various aspects of regular physical activity. An initial draft questionnaire was prepared focusing on the specific and general issues identified. The initial questionnaire underwent a review by a panel of 5 experts from Public Health (NCDs and Adolescent Health), Health Administration, Health Psychology, Social Sciences, and Epidemiology, who made necessary corrections. The questionnaire underwent pre-testing among 40 students and again presented to the expert panel with modifications for finalization. Validity of the questionnaire during the review and refinement phase was assessed by the measurement of Content Validity Index (CVI) of each of the construct-wise sub-scales (i.e., domain-wise). The different scales in the final questionnaire had CVIs ranging between, 0.88–0.95 (Sub-scales: behavioral belief – 0.92, evaluation of behavioral outcome – 0.88, normative beliefs – 0.88, motivation to comply – 0.92, control beliefs – 0.90, perceived power – 0.95). Based on the prior experience of working with adolescents on similar issues, and the experts’ opinion there was a unanimous consensus for dichotomous responses to each question for better comprehensibility of questions and clear discrimination on the intender – non-intender spectrum. The internal consistency reliability of the scales was gauged using Cronbach’s alpha, yielding values ranging between 0.68 and 0.81 (Sub-scales: behavioral belief – 0.80, evaluation of behavioral outcome – 0.82, normative beliefs – 0.68, motivation to comply – 0.74, control beliefs – 0.78, perceived power – 0.81).

Building on the construct-wise insights gleaned from elicitation interviews exploring physical activity performance and practices, the intervention booklet and demonstration materials were designed, developed, and subjected to pre-testing. The conclusions derived from the pre-intervention survey played a pivotal role in refining the intervention tools’ content. Within the intervention booklet, participants accessed informative content on physical activity, replete with concrete examples of Moderate and Vigorous intensity physical activities, while emphasizing the significance of cultivating and sustaining the habit of regular physical activity. The booklet provided information on benefits of regular physical activity, e.g., maintaining physical and mental health, weight management, reduced risk of various chronic diseases, and improved concentration and focus, etc. It also encouraged the adolescents to start regular physical activity even if they felt it was too late. Engaging in sports activities were also described as a means to regular physical activity habits. The content also focused on initiating dialogues for available spaces for performing physical activities, in schools as well as in the community areas. Alongside, the booklet provided information regarding the myths and misconceptions regarding physical activities particularly focusing on subjective norms and perceived behavioral control.

Dynamic interactive sessions, allowing for intra-group discussions and group questions along with the interactions with the intervention implementer provided a platform for in-depth discussions, delving into common misconceptions and beliefs pertaining to physical activity engagement. The interventions were tailored to focus on fostering enablers, facilitating the adoption of regular physical activity, while concurrently addressing barriers that could impede progress. Normative influencers and their pivotal role in motivating participants to embrace physical activity were highlighted, underscoring their potential impact.

In close collaboration with subject experts, the intervention tools were prepared, adhering to established guidelines governing physical activity practices. The comprehensive approach to intervention development, bolstered by pre-testing and expert input, ensured the efficacy of the tools in driving positive behavioral change towards regular physical activity among the participants.

### 2.5 Techniques

Baseline data were collected from students in grades VII to X, encompassing both the intervention and control groups, utilizing a self-administered questionnaire, after securing their assent. To ensure data integration during intervention, and follow-up, each participant was assigned an Identification (ID) code, by a team independent of the school teachers or the researchers. At the time of recruitment in the study and obtaining assent, the participants were explained the role of the id number, and how the id number will be used to keep their responses blinded from the teachers and also the research team. The use of id was done for ensuring blinded response files, to minimize socially desirable responses. Subsequent to the baseline data collection, the intervention was specifically administered to the intervention group, adopting a grade-wise approach, i.e., intervention was provided to the selected section on one grade at a time.

The distribution of instructive booklets among participants preceded informative lectures, thoughtfully anchored in the booklet’s contents. To enhance comprehension, pre-designed computer-based slides were employed as visual aids, effectively highlighting the existing disparities between their thoughts and actions. Interactive sessions and discussions were woven into the interventions, fostering an environment conducive to active participation and engagement. The interactive sessions provided scope for discussion among peers and coming with a group questions, along with the individual-level interactions with the intervention implementers. The whole of the intervention package comprising booklets, lectures, and interactions were standardized through experts’ reviews and pre-testing. The standardization ensured minimal difference when implementing intervention in different grades in the intervention school. Participants were encouraged to seek clarifications during a dedicated question-and-answer session, effectively addressing queries and doubts. All the participants of the intervention group included in the final analyses were provided with the booklet and all of them attended the lectures and interactive sessions. During the interactive sessions, all the participants interacted at least once, while the mean (SD) number of questions asked by each participant was 1.7 (0.5). Out of all the questions asked during interaction, 64.6% questions were directly related to the content of the booklet and the lectures, while 8.4% were related to the contents of the booklet only, 21.3% regarding the lecture contents, and rest related to the content of the interactions. It was noted that the students were mostly concerned regarding whether they will be able to perform regular physical activities given their different constraints, and some raised concerns regarding the sometimes-conflicting social norms on putting studies above physical fitness.

Three months after the intervention’s conclusion, follow-up data collection ensued, gauging the lasting impact of the interventions. The same questionnaire was administered to the control group during follow-up, mirroring the approach employed in the intervention group, albeit without any specific intervention during the interim period. Upon the completion of follow-up data collection, the control group also received health education interventions during the study period, following the same methodical approach applied in the intervention group. This study design aimed to yield reliable and robust outcomes, exploring the potential impact of health education interventions in fostering positive behavioral change. By administering tailored interventions and collecting follow-up data, the study endeavors to shed light on the transformative potential of health education, paving the way towards embracing regular physical activity and fostering improved well-being among the participants.

### 2.6 Statistical analysis

The pre-intervention and post-intervention data were linked in a spreadsheet using unique codes, and the resulting cleaned dataset was subjected to statistical analysis using Statistical Package for Social Sciences (SPSS) software version 21 (IBM, Chicago, IL, USA). To facilitate computation, items were coded as 1 or 2, with 2 indicating a favorable response towards healthier habits. Negatively framed items were reverse-coded while maintaining the scoring directionality, ensuring higher scores for favorable responses. Construct scores were calculated by multiplying the scores for the contributing domains, such as ‘Attitude,’ which was derived from the Behavioral belief score and evaluation of behavioral outcome score. Similar calculations were performed for ‘Subjective Norm’ and ‘Perceived Behavioral Control.’ A linear combination of item set responses yielded individual domain-specific scores. To identify ‘Intenders,’ representing individuals with the latent variable ‘Behavioral intention,’ a two-step cluster analysis with maximum likelihood estimation was conducted, utilizing calculated Attitude, Subjective norm, and Perceived behavioral control scores separately for the pre-intervention and post-intervention phases, encompassing both the intervention and control groups. Participants with higher mean scores in the constructs were categorized as ‘Intenders,’ while the remaining were labeled as ‘Non-intenders’ (refer to Table 2). The cluster analyses were deemed statistically adequate using the Silhouette measure. Item-specific responses and proportions of intenders were compared between the pre-intervention and post-intervention phases in both study groups. By employing this comprehensive approach to data analysis, the study seeks to illuminate potential changes in attitudes, subjective norms, and perceived behavioral control, and their implications on behavioral intention following the interventions, providing valuable insights for targeted strategies to foster positive health habits.

**Table 2:** Description of clusters in Pre-intervention and post-intervention phases as per the defining constructs.

**Model constructs**	**Pre-intervention**^**a**^		**Post-Intervention**^**a**^	
	**Cluster 1:** **Higher intention [*n* = 169]**	**Cluster 2:** **Lower intention [*n* = 82]**	**Cluster 1:** **Higher intention [*n* = 127]**	**Cluster 2:** **Lower intention [*n* = 124]**
Attitude	120.38 (±16.45)	92.20 (±15.97)	122.39 (±13.73)	100.70 (±24.79)
Subjective norm	152.54 (±27.45)	115.07 (±16.02)	175.82 (±19.57)	119.15 (±26.12)
Perceived behavioral control	102.31 (±23.46)	113.59 (±13.67)	122.28 (±31.30)	103.03 (±18.36)

‘*n*’ indicates the number of participants in each identified cluster, and values within parentheses in each cell represent the respective standard deviations. ^a^No outliers were detected; the average silhouette measure of cluster cohesion and separation was 0.5.

Statistically significant differences in items between the two study groups before and after the intervention phase were assessed using the chi-square test. Within each study group, construct scores were compared using paired *t*-tests based on intention clusters and observation points. The intervention’s effect was quantified by Relative Risk (RR) for being in the higher intention cluster. For individual-level models, the participants’ id was considered to link the pre-intervention and post-intervention responses. A Generalized Linear Model (GLM) with a log-linear link and Poisson distribution assumptions was employed to measure the RR, incorporating robust standard errors. The GLM was adjusted for gender, baseline (pre-intervention) intention cluster, and their interaction with the receipt of intervention (i.e., study groups). The model demonstrated statistical significance (*P*_χ2_ < 0.001). In all statistical analyses, a two-tailed *p*-value of ≤0.05 was considered statistically significant.

### 2.7 Ethical consideration

The study received approval from the Institutional Ethics Committee of the All India Institute of Hygiene and Public Health, Kolkata. Prior to data collection, permission was obtained from the head of each school. Informed written assent was then sought from the participants, while informed written consent was obtained from their guardians.

## 3 Results

### 3.1 Measurement of intention toward performing regular physical activity

The pre- and post-intervention comparison of items in the two contributing domains of ‘Attitude’ construct is depicted in Table 3. In the intervention group, there was a statistically significant post-intervention difference with improvement in post-intervention proportion to 88.98% from 71.19% in the baseline regarding favorable behavioral belief on the idea that regular physical activity helps boost energy levels throughout the day. However, in the control group the proportions were comparable at the baseline and at the follow-up. Similarly, regarding evaluation of behavioral outcome on this item the proportions at baseline and follow-up were comparable in intervention and control group. On the other hand, in the control group there was a statistically significant decrease in the proportion of respondents, from 49.62% in the baseline to 28.57% in the follow-up, with behavioral belief regarding decrease in sleeping time with regular physical activity. In the intervention group the difference was not statistically significant.

**Table 3:** Pre- and post-intervention comparison of attitudes regarding regular physical activity among intervention and control groups.

**Items**	**Response**	**Intervention (*n* = 118)**			**Control (*n* = 133)**		
		**Pre-intervention**	**Post-intervention**	***P*-value, χ**^**2**^	**Pre-intervention**	**Post-intervention**	***P*-value, χ**^**2**^
Proper functioning of body system	Behavioral beliefs^a^	111 (94.07)	110 (93.22)	0.790, 0.07	123 (92.48)	126 (94.74)	0.452, 0.57
	Evaluation of behavioral outcome	110 (93.22)	103 (87.29)	0.124, 2.36	128 (96.24)	129 (96.99)	0.735, 0.11
Boosting energy level throughout the day	Behavioral beliefs	84 (71.19)	105 (88.98)	0.001, 11.72	117 (87.97)	119 (89.47)	0.698, 0.15
	Evaluation of behavioral outcome	98 (83.05)	94 (79.66)	0.504, 0.45	112 (84.21)	113 (84.96)	0.865, 0.03
Increasing laziness	Behavioral beliefs^a^	85 (72.03)	84 (71.19)	0.885, 0.02	97 (72.93)	104 (78.19)	0.380, 1.93
	Evaluation of behavioral outcome	94 (79.67)	81 (68.64)	0.053, 3.74	109 (81.95)	109 (81.95)	1.000, 0.00
Decreasing sleeping time	Behavioral beliefs	71 (60.17)	58 (49.15)	0.089, 2.89	66 (49.62)	38 (28.57)	0.000, 12.38
	Evaluation of behavioral outcome	75 (63.56)	64 (54.24)	0.146, 2.12	81 (60.90)	85 (63.91)	0.613, 0.26
Increasing hunger	Behavioral beliefs	80 (67.80)	82 (69.49)	0.779, 0.08	109 (81.95)	107 (80.45)	0.754, 0.10
	Evaluation of behavioral outcome	63 (53.39)	68 (57.63)	0.513, 0.43	73 (54.89)	87 (65.41)	0.080, 3.07
Increase in strength and height	Behavioral beliefs	68 (57.63)	62 (52.54)	0.432, 0.62	100 (75.19)	112 (84.21)	0.067, 3.35
	Evaluation of behavioral outcome	98 (83.05)	99 (83.90)	0.861, 0.03	120 (90.23)	119 (89.47)	0.839, 0.04

‘*n*’ represents the number of completed responses in each group. Values within parentheses represent respective column percentages. *P*-values calculated by chi-squared test. ^**a**^Items framed in negative direction (i.e., a lower proportion is favorable).

Table 4 describes the pre- and post-intervention comparison of items in the two contributing domains of ‘Subjective Norms’ construct. Among the intervention group participants, relatives and other family members emerged as an important normative influencer variable, as there was a statistically significant decrease in the proportion of the respondents at baseline and follow-up for both the domains, i.e., normative beliefs (60.17 to 38.98%) and motivation to comply (72.03 to 52.54%). However, in the control group there was a statistically significant increase in the proportion of respondents with motivation to comply to relatives and other family members (43.61 to 69.17%), and social media contents (61.65–77.44%). In the control group there was statistically significant increase in the proportion with motivation to comply to father and mother, however in the intervention group despite a higher proportion at both the phases the trend was not statistically significant. In these cases, the differences in normative beliefs were comparable at baseline and follow-up, and hence, not statistically significant.

**Table 4:** Pre- and post-intervention comparison of subjective norm regarding regular physical activity among intervention and control groups.

**Items**	**Response**	**Intervention (*n* = 118)**			**Control (*n* = 133)**		
		**Pre-intervention**	**Post-intervention**	***P*-value, χ**^**2**^	**Pre-intervention**	**Post-intervention**	***P*-value, χ**^**2**^
Mother	Normative beliefs	98 (83.05)	99 (83.90)	0.861, 0.03	127 (95.49)	127 (95.49)	1.000, 0.00
	Motivation to comply	99 (83.90)	97 (82.20)	0.729, 0.12	107 (80.45)	124 (93.23)	0.002, 9.51
Father	Normative beliefs	99 (83.90)	90 (76.27)	0.142, 2.15	118 (88.72)	111 (83.46)	0.215, 1.54
	Motivation to comply	93 (78.81)	94 (79.66)	0.872, 0.03	95 (71.43)	116 (87.22)	0.001, 10.11
Relatives/other family members	Normative beliefs	71 (60.17)	46 (38.98)	0.001, 10.59	85 (63.91)	88 (66.17)	0.700, 0.15
	Motivation to comply^a^	85 (72.03)	62 (52.54)	0.002, 9.54	58 (43.61)	92 (69.17)	0.000, 17.67
Friends/peers	Normative beliefs	82 (69.49)	93 (78.81)	0.102, 2.67	90 (67.67)	105 (78.95)	0.038, 4.32
	Motivation to comply^a^	83 (70.34)	75 (63.56)	0.156, 3.72	83 (62.41)	98 (73.68)	0.049, 3.89
Teachers	Normative beliefs	90 (76.27)	90 (76.27)	1.000, 0.00	109 (81.95)	118 (88.72)	0.119, 2.43
	Motivation to comply	88 (74.58)	78 (66.10)	0.115, 4.33	91 (68.42)	113 (84.96)	0.001, 10.18
Contents of television	Normative beliefs	57 (48.31)	57 (48.31)	1.000, 0.00	69 (51.88)	83 (62.41)	0.083, 3.00
	Motivation to comply	68 (57.63)	54 (45.76)	0.068, 3.33	91 (68.42)	100 (75.19)	0.220, 1.50
Contents/discussions in Social media	Normative beliefs	44 (37.29)	39 (33.05)	0.495, 0.46	61 (45.86)	68 (51.13)	0.390, 0.74
	Motivation to comply	85 (72.03)	88 (74.58)	0.659, 0.19	82 (61.65)	103 (77.44)	0.005, 7.83

‘*n*’ represents the number of completed responses in each group. Values within parentheses represent respective column percentages. *P*-values calculated by chi-squared test. ^**a**^Items framed in negative direction (i.e., a lower proportion is favorable).

The baseline and follow-up phases comparison of perceived behavioral control in the two study groups are shown in Table 5. In the intervention group there was an improvement in the perceived power regarding choosing to perform regular physical activity even when there is no playground nearby (51.69–78.81%), even when there is pressure of homework and academic work (39.83–60.17%), and even during special days/occasions (40.68–64.41%). In control group, there was an increase in the proportion of perceived power in the follow-up phase regarding performing regular physical activity during special days/occasions (42.86–57.14%). On the other hand, in the intervention group, a favorable change in control belief in the follow-up phase was noted by a decrease in proportion (negatively framed questions) for the items, choosing to play outdoors despite homework and academic pressure (41.52–17.80%), and choosing to perform physical activity even during special days/occasions (51.69–28.21%). For these items, the change in the follow-up phase in the control group was not favorable, as there was a statistically significant increase in proportion of control beliefs for these items.

**Table 5:** Pre- and post-intervention comparison of perceived behavioral control regarding regular physical activity among intervention and control groups.

**Items**	**Response**	**Intervention (*n* = 118)**			**Control (*n* = 133)**		
		**Pre-intervention**	**Post-intervention**	***P*-value, χ**^**2**^	**Pre-intervention**	**Post-intervention**	***P*-value, χ**^**2**^
Choosing to perform regular physical activity even when there is no playground nearby	Control belief^a^	19 (16.10)	23 (19.49)	0.496, 0.46	53 (39.85)	61 (45.86)	0.322, 0.98
	Perceived power	61 (51.69)	93 (78.81)	0.000, 19.14	108 (81.20)	95 (71.43)	0.061, 3.51
Choosing to play outdoors despite homework and academic pressure	Control belief^a^	49 (41.52)	21 (17.80)	0.000, 15.92	39 (29.32)	59 (44.36)	0.011, 6.46
	Perceived power	47 (39.83)	71 (60.17)	0.002, 9.76	59 (44.36)	64 (48.12)	0.539, 0.38
Choosing to perform regular physical activity despite the thought that people may tease an obese person in a very embarrassing manner	Control belief	46 (38.98)	57 (48.31)	0.149, 2.08	51 (38.34)	66 (49.62)	0.064, 3.43
	Perceived power	88 (74.58)	90 (76.27)	0.762, 0.09	83 (62.41)	89 (66.92)	0.442, 0.59
Choosing to perform regular physical activity even if playing outdoor games become difficult (e.g., in rainy season)	Control belief	30 (25.42)	40 (33.90)	0.154, 2.03	41 (30.83)	45 (33.83)	0.600, 0.27
	Perceived power	50 (42.37)	62 (52.54)	0.118, 2.45	75 (56.39)	68 (51.13)	0.389, 0.74
Choosing to play or do physical activity despite video games and/or mobile phone games	Control belief	38 (32.20)	47 (39.83)	0.222, 1.49	72 (54.14)	66 (49.62)	0.462, 0.54
	Perceived power	68 (57.63)	69 (58.47)	0.895, 0.02	88 (66.17)	79 (59.40)	0.254, 1.30
Choosing to perform physical activity even during special days/occasions	Control belief ^a^	61 (51.69)	34 (28.81)	0.000, 12.84	54 (40.60)	72 (54.14)	0.027, 4.89
	Perceived power	48 (40.68)	76 (64.41)	0.000, 13.32	57 (42.86)	76 (57.14)	0.020, 5.43
Choosing to do regular physical activity/exercises despite laziness	Control belief	66 (55.93)	70 (59.32)	0.598, 0.28	53 (39.85)	42 (31.58)	0.159, 1.98
	Perceived power	65 (55.08)	76 (64.41)	0.144, 2.13	89 (66.92)	100 (75.19)	0.137, 2.12

‘*n*’ represents the number of completed responses in each group. Values within parentheses represent respective column percentages. *P*-values calculated by chi-squared test. ^**a**^Items framed in negative direction (i.e., a lower proportion is favorable).

### 3.2 Intenders and non-intenders of regular physical activity

**Figure 2:**
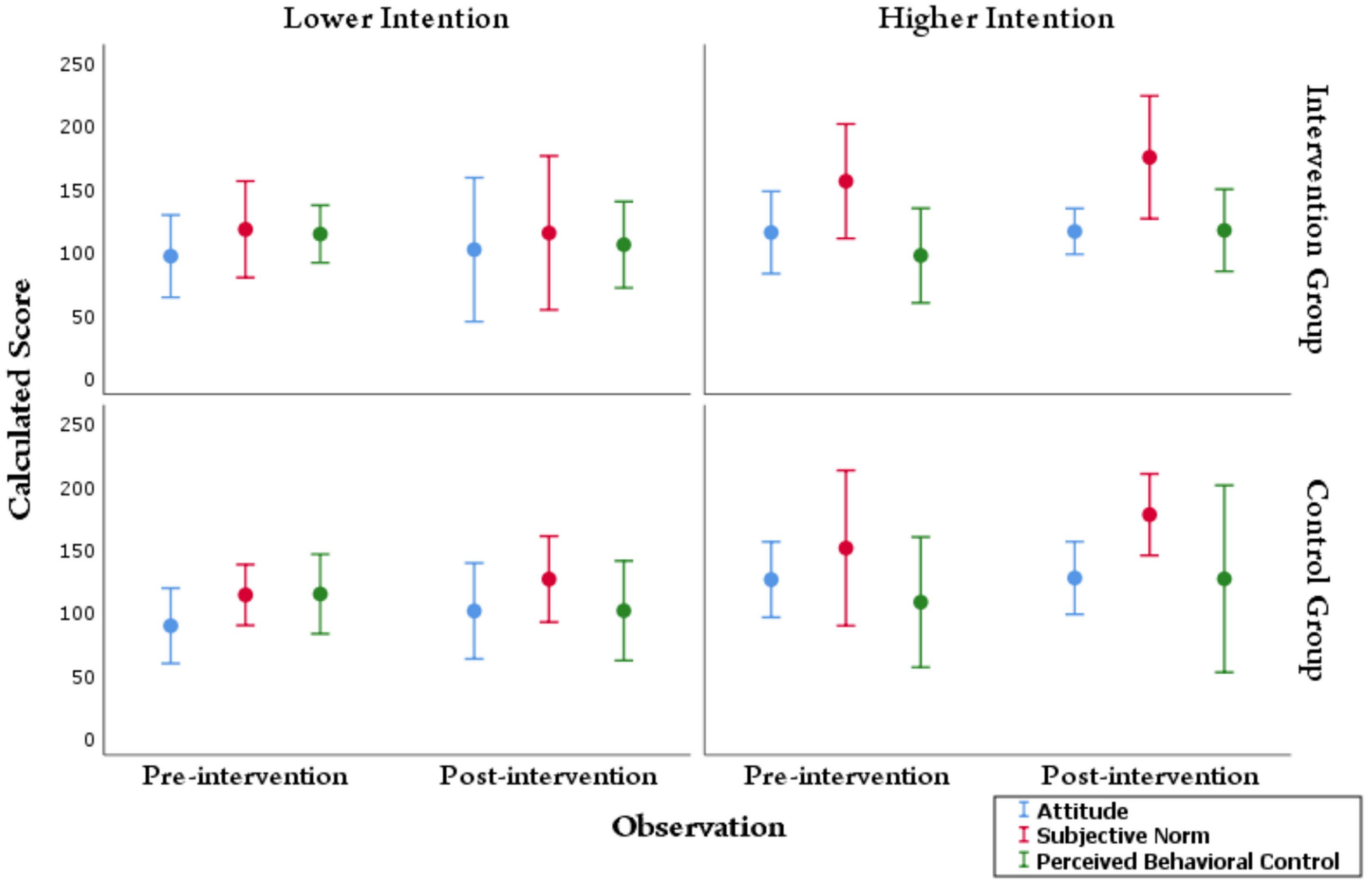
Construct-wise score distribution of the study groups according to higher and lower intention clusters during pre- and post-intervention phases. The circles indicate the mean value computed, and the bars represent two standard deviations (2SD) around the mean. The lower intention and higher intention clusters were calculated independently for pre-intervention and post-intervention responses. The comparison of mean scores at the pre-and post-intervention phases was made with the help of an independent sample *t*-test. No statistically significant difference was observed in the intervention or control groups when comparing the scores in the pre-intervention lower intention cluster with the post-intervention lower intention cluster. However, in the intervention group, there was a significant improvement in the mean score of subjective norm and perceived behavioral control following intervention in the higher intention cluster.

The construct-wise score comparison of baseline and follow-up phases in the study groups is provided in Table 6. The construct-wise scores improved following the intervention in the intervention group, with a statistically significant improvement observed in the intervention group for perceived behavioral control [102.25 (±18.43) to 109.36 (±17.62)]. The differences in mean scores for perceived behavioral control showed an increasing trend in the control group as well, but was not statistically significant. Subjective norm mean scores were statistically higher in the follow-up phase in the control group [138.79 (± 31.54) to 155.59 (± 30.19)], while in the intervention group they were comparable and hence, not found to be statistically significant. Figure 2 compares construct-wise scores before and after intervention in the study groups according to the intention clusters. Although the mean scores indicate a comparatively better baseline characteristics of the control group, the intention cluster-wise (i.e., categorical) comparisons establish a rather comparable baseline among the two study groups.

**Table 6:** Pre- and post-intervention comparison of construct-wise mean scores regarding regular physical activity among intervention and control groups.

**Model constructs**	**Intervention (*n* = 118)**			**Control (*n* = 133)**		
	**Pre-intervention**	**Post-intervention**	***P*-value** **(t, df)**	**Pre-intervention**	**Post-intervention**	***P*-value** **(t, df)**
Attitude	108.07 (±18.59)	106.63 (±24.01)	0.602 (0.52, 117)	113.93 (±22.60)	115.66 (±20.88)	0.519 (−0.65, 132)
Subjective norm	142.01 (±28.18)	137.64 (±40.73)	0.285 (1.07, 117)	138.79 (±31.54)	155.59 (±30.19)	0.000 (−4.49, 132)
Perceived behavioral control	102.25 (±18.43)	109.36 (±17.62)	0.006 (−2.82, 117)	109.32 (± 23.31)	115.37 (±33.44)	0.078 (−1.77, 132)

‘*n*’ represents the number of completed responses in each group. Values within parentheses represent respective standard deviations. *P*-values calculated by paired *t*-test. *t*, *t*-statistic; df, degrees of freedom.

### 3.3 Effect of intervention

It was noted that boys had a higher chance of becoming intender for regular physical activity (RR: 1.13, 95% CI: 1.00–1.27). However, the intention of the participants measured at the baseline, i.e., before the intervention did not have a statistically significant effect on becoming an intender. Controlling for these factors and the different interactions, it was noted that the intervention had a statistically significant effect among the study participants towards becoming intender. The Intervention group documented an RR of 1.24 (1.04–1.48) compared to the Control group, for becoming an intender towards regular physical activity in the follow-up phase. Table 7 represents the generalized linear model showing the effect of intervention in improving intention.

**Table 7:** General linear model showing different factors of improved intention for performing regular physical activity.

**Factors for becoming an intender for regular physical activity**	**aRR (95% CI)**	***P*-value**
Intervention (Ref.: Control group)		
Intervention Group	1.24 (1.04–1.48)	0.018
Pre-Intervention Intention (Ref.: High Intention Cluster)		
Low Intention Cluster	0.98 (0.87–1.11)	0.769
Gender (Ref.: Girls)		
Boys	1.13 (1.00–1.27)	0.043

aRR, Adjusted Relative Risk; CI, Confidence interval; *P*-values obtained through Generalized Linear Model (GLM) with the log-linear link under Poisson distribution function and robust standard errors: Akaike’s Information Criterion (AIC) and Bayesian Information Criterion (BIC) were 630.366 and 651.518 respectively, with the scale parameter for the model set at 1. The model has been adjusted for the interaction between study groups and the pre-intervention cluster, with model intercept: 1.35 (95% CI: 1.19–1.5).

## 4 Discussion

### 4.1 Findings in light of relevant literature

In the present study, within the intervention group, there was a notable increase in the proportion of agreement for the item “Regular physical activity helps to boost energy level throughout the day” following the intervention, and this difference was statistically significant. Consequently, the belief among students that regular physical activity is advantageous and can enhance energy levels witnessed a noteworthy improvement after the intervention. The current results align with Poobalan et al.’s report, wherein 66% of the participants perceived regular exercise as promoting health, while approximately 20–30% regarded exercise as easy, relaxing, and enjoyable (31). Kumar et al. conducted a study that revealed participants with a sound understanding of cardiovascular disease (CVD) were more inclined to engage in at least 1 h of daily exercise (23). Within the intervention group, there was a marginal reduction from 108.07 (±18.59) to 106.63 (±24.01) in the mean score of ‘Attitude’ towards regular physical activity during the post-intervention phase, although this difference was not statistically significant. Interestingly, this finding contrasts with the results from a study conducted in Iran by Darabi et al., where they reported a significantly higher mean score of ‘Attitude’ in the experimental group (22). The contrast in the evidence might be possible due to the difference in study populations and study designs, as in the relevant literature, only girls were considered in the study. Still, in the current study, both boys and girls were included. Within the intervention group, the mean score of ‘Subjective Norm’ for regular physical activity experienced a decrease after the intervention, but this difference was also not statistically significant. These outcomes may be attributed to the influence of various external factors. However, considering the current study’s objective the proportions of participants as per intervention cluster provides a better understanding, although the intervention clusters were arrived at considering all the construct-wise scores, as mentioned earlier. Interestingly, our study’s findings in the intervention group diverged from those of Darabi et al., who reported a significantly higher mean score of ‘Subjective Norm’ in the experimental group (22). Didarloo et al. observed a statistically significant change in the mean score of ‘subjective norms’ between the two groups after the intervention (24). Similarly, in an interventional study carried out among Iranian adolescents by Mazloomy-Mahmoodabad et al., the results were consistent with the aforementioned finding (25).

Within the intervention group, there was a notable improvement towards performing regular physical activity despite homework and academic pressure making it difficult to go and play outside. After the intervention, the awareness of outdoor games and adequate outdoor physical activities increased. Hence, participants realized and believed that though it would be difficult for them to go and play outside due to time constraints and study pressure, still they would do it. This finding was similar to the finding observed in the cross-sectional study done in India among students by Tomy et al., who reported that lack of time was the most critical barrier to physical activity (17). Barriers to participation in physical activity, being time constraints, tiredness, stress, family control, lack of support and motivation, and safety issues, were observed in different pieces of literature (18, 34). The most frequent barriers identified in a systematic review of systematic reviews were the lack of time and support from peer groups, family members, and teachers (35). Silva et al. stated in their systematic review that the main barriers identified among students were lack of time, motivation, and accessible places (36). A recent study among English adolescents documented more than 50 different barriers for performing regular physical activity. As per the authors apart from academic pressure, laziness, lack of motivators, etc. certain negative experiences, and often anthropometric status plays an important part among the students for not taking up physical activity regularly (32). The current study did not directly seek the practices or their barriers, but documented the intention towards the same, and the students’ willingness given different constraining circumstances. The current findings do support these different barriers reported by Moore et al., except the issues of psychological factors and anthropometric barriers, which, were not studied here (32).

Schools play a crucial role in fostering regular physical activity among students through the implementation of comprehensive school-based physical activity programs. These programs may include structured physical education classes, physical activity breaks, and initiatives such as ‘walk/bicycle to school’ campaigns. By providing motivation and creating opportunities for physical activity, schools can contribute significantly to the overall well-being and health of their students. All schools should have a playground or adequate space for physical activities. Most of the intervention group participants during post-intervention were confident to play outside despite academic pressure, although they believed that homework and academic pressure made it difficult. The intervention package improved their confidence and self-control over performing the activity. The ‘Perceived behavioral control’ mean score for regular physical activity increased from 102.25 (±18.43) to 109.36 (±17.62) in the Intervention group after the intervention, and the difference was statistically significant. The finding proved that the intervention package effectively improved the perceived control over regular physical activity in the current study. The RR of becoming an intender for regular physical activity in the Intervention group was 1.24 (1.04–1.48) when compared to the Control group. These findings from the current study are consistent with the result from the study done by Darabi et al. (22) The application of digital technology can play a very integral role in motivating and shaping the habits of students in performing regular physical activity (37, 38).

### 4.2 Limitations

Apart from the constructs and items in the TPB, changes in intention status are influenced by various external and environmental factors. However, due to the focused application of the model framework, these exogenous variables were not considered in the study. Furthermore, as the participants self-reported their information on the constructs, there is a potential for social desirability bias, even with the assurance of confidentiality measures. The controlled design with comparable groups, however, firmly alleviates the concern of the observed effect being attributed to any form of social desirability or respondent biases. It was noted that based on the baseline construct-wise scores, the control group was, in some cases, better in comparison to the intervention group. This apparent discrepancy wanes of when clusters of higher and lower intention are considered, which has been done in the case of the generalized linear model to document the intervention effect.

## 5 Conclusion

Schools cover a critical period for establishing healthy behaviors and are a unique setting for promoting healthy behavioral practices. Health Promoting Schools has been recognized as a strategic and cost-effective vehicle to promote positive development and health and can effectively reduce the emergence of a significant NCDs risk factor like physical inactivity. Behavioral intention, the primary predictor of behavior utilized in the interventions of this study, showed a statistically significant improvement in ‘perceived behavioral control’ regarding physical activity among the intervention group during the post-intervention phase. This enhancement in intention indicates a positive response to the intervention’s effectiveness. Based on the evidence, scientific, effective, and appropriate behavioral change intervention can catalyze positive changes in their intention and behavior. School children should have the accessibility to adequate space for performing physical activities. In the context of our previous study on the effectiveness of a health promotion intervention regarding intentions towards consumption of healthy diet (33), the current research study provides valuable insights into the impact of a similar intervention on behavioral intentions toward regular physical activity among adolescents. Our findings contribute to the understanding of effective strategies to enhance adolescents’ motivation for regular physical activity, potentially guiding future interventions and policies aimed at promoting healthier behaviors during this crucial developmental stage. These results are complementary to our findings of healthy diet intentions, and together should be adopted for effective intervention. To ensure effective decision-making and address issues of concern, stakeholder participation, including parent-teacher meetings, becomes essential. Schools should actively engage in cross-sectoral partnerships with a diverse group of stakeholders, as they can offer valuable insights and collaborate on solutions to tackle barriers that extend beyond the school environment. By fostering strong partnerships, schools can better facilitate control over challenges and enhance overall effectiveness in addressing critical issues. In conclusion, these meticulously tailored interventions hold great promise in fostering a healthier and more proactive cohort, equipped to embrace regular physical activity with unwavering resolve.

## Data availability statement

The original contributions presented in the study are included in the article/supplementary material, further inquiries can be directed to the corresponding author.

## Ethics statement

The studies involving humans were approved by Institutional Ethics Committee, All India Institute of Hygiene and Public Health, Kolkata. The studies were conducted in accordance with the local legislation and institutional requirements. Written informed consent for participation in this study was provided by the participants’ legal guardians/next of kin.

## Author contributions

SJ: Writing – review & editing, Writing – original draft, Visualization, Validation, Software, Resources, Project administration, Methodology, Investigation, Formal analysis, Data curation, Conceptualization. MD: Writing – review & editing, Writing – original draft, Supervision, Project administration, Methodology, Conceptualization. CT: Writing – original draft, Supervision, Methodology, Investigation, Conceptualization. AL: Writing – original draft, Writing – review & editing, Visualization, Validation, Supervision, Software, Resources, Methodology, Formal analysis.
